# Case reports of collision and composite carcinomas of the thyroid: an insight into their origin and clinical significance

**DOI:** 10.1186/s12902-023-01409-z

**Published:** 2023-08-14

**Authors:** Tao Ma, Ruixiao Wang, Xu Zhou, Liqiang Liu, Aijing Pan, Hongmei Wang, Lingyan Huang

**Affiliations:** 1 Surgical Oncology, General Hospital of Ningxia Medical University, Yinchuan, 750000 Ningxia Province China; 2 Pathological Department, General Hospital of Ningxia Medical University, No.804 Shengli South Street, Xingqing District, Yinchuan, 750000 Ningxia Province China; 3 Ultrosound Department, General Hospital of Ningxia Medical University, Ningxia Province, Yinchuan, 750000 China; 4 Clinical Medical College, Ningxia Medical University, Yinchuan, 750000 Ningxia Province China; 5 Department of Pharmacology, School of Medicine, Southeast University, Dingjiaqiao 87, Nanjing, 210009 Jiangsu China

**Keywords:** Case report, Collision carcinoma, Composite carcinoma, Thyroid

## Abstract

**Background:**

Collision and composite carcinomas of the thyroid are extremely rare, and their clinical and biological characteristics are poorly understood.

**Case presentation:**

The first case was a 41-year-old female patient with a right thyroid nodule. Pathological diagnosis was papillary thyroid carcinoma (PTC) and medullary thyroid carcinoma composite carcinoma. Surgical treatment was right thyroid lobectomy + left partial thyroidectomy + right central neck lymph node dissection. The second case was a 60-year-old female with bilateral thyroid nodules. Total thyroidectomy was performed, and the pathological diagnosis was thyroid collision carcinoma involving follicular thyroid carcinoma on the left side and PTC on the right side.

**Summary:**

The clinical, histological and gene changes of collision and composite carcinomas of the thyroid are poorly described. With different biological invasion characteristics, the ideal treatment and the prognosis is currently unknown and individualized treatment is necessary.

**Conclusions:**

It is recommended that in composite carcinoma, each cancer is evaluated and treated according to the most severe tumor. Collision carcinoma should be treated as two separate synchronous primary tumors. For both collision and composite carcinomas of the thyroid, the follow-up after treatment should be extensive.

## Background

The incidence of thyroid cancer is increasing worldwide. In China, the incidence of thyroid cancer in women is higher than in men, and the ratio of male to female patients is about 1:3.8 in urban areas [1]. As the most common malignant tumor of the endocrine system, the common pathological types of thyroid cancer include papillary carcinoma, follicular carcinoma, medullary carcinoma, and undifferentiated carcinoma. Papillary carcinomas account for more than 85% of all thyroid cancers [1], and they often occur in the unilateral or bilateral lobes of the thyroid gland as a single pathological type.

Although it is rare that more than one pathological type of thyroid carcinoma occurs simultaneously, composite carcinomas and collision carcinomas of the thyroid occur occasionally. Collision carcinomas and composite carcinomas account for only about 1% of all malignant thyroid tumors [1]. A composite tumor is one in which two discrete but closely adjacent cell groups in the same tumor focus individually express thyroglobulin and calcitonin [2]. Collision carcinomas also involve two types of tumor foci in the same organ, but the two lesions exist independently, with normal tissue between them and without mixing [3]. Although similar, composite and collisional carcinomas of the thyroid have unique clinical and biological characteristics.

We collected more than 2000 patients with thyroid cancer from a single medical center and we report here one case of thyroid composite cancer and one case of thyroid collision cancer. The differences in clinical and pathological features, gene changes, therapeutic effects, and prognosis between thyroid composite carcinomas and collision carcinomas were investigated by reviewing the literature.

### Case presentations

**Fig. 1:**
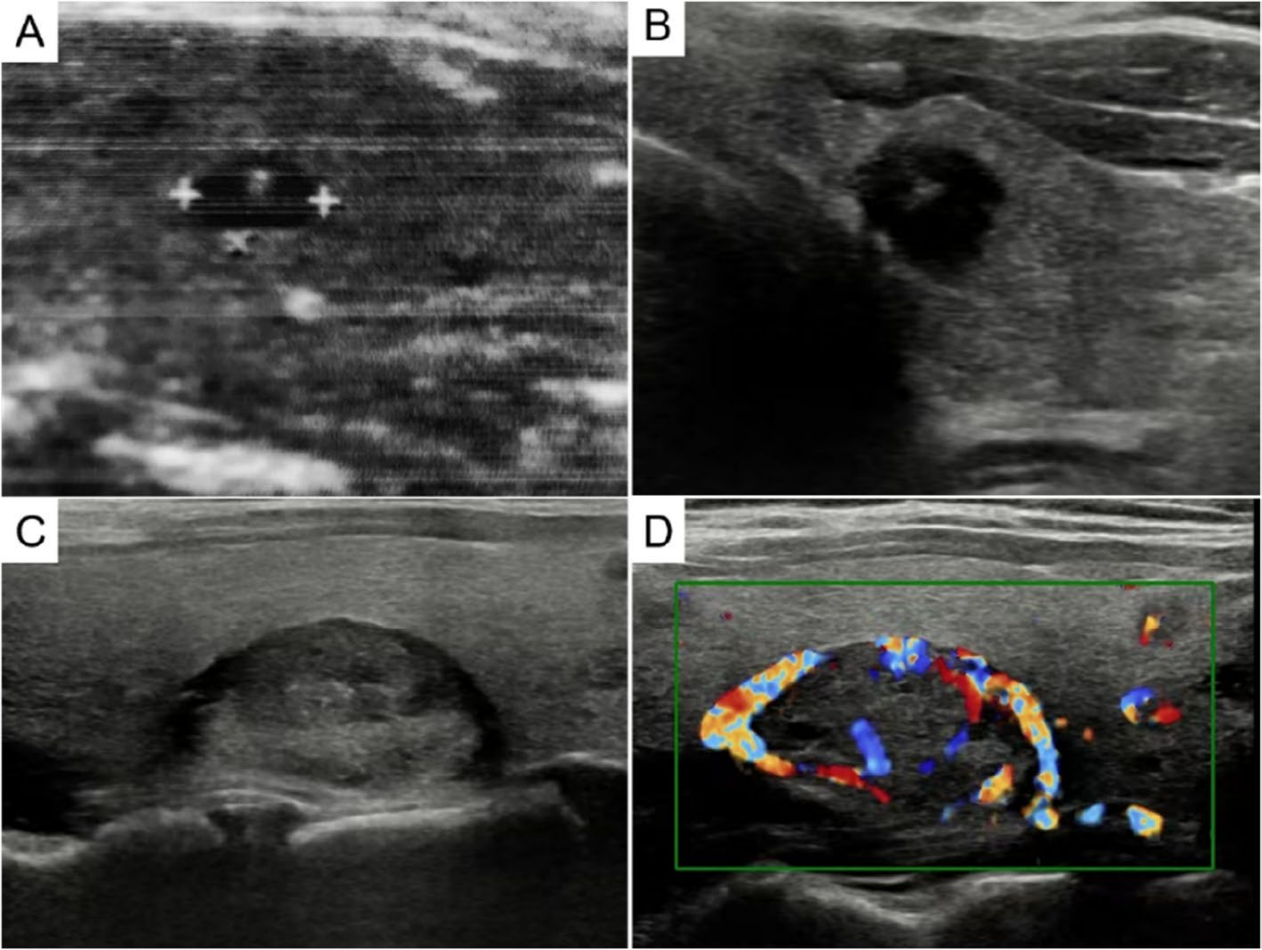
B ultrasound showed the thyroid collision and composite carcinomas. **A** Thyroid collision carcinoma showed a hypoechoic solid mass with unclear borders, uneven internal echo, and sand-like calcification. **B-D** Thyroid composite carcinoma presented a solid nodule on the right side of the thyroid gland that was extremely hypoechoic, with unclear boundary and irregular shape, aspect ratio greater than 1, and edge angulation (**B**). **C** The left thyroid nodule had clear boundary, regular shape, smooth capsule, lateral sound attenuation, and mixed echo inside. **D** The blood flow signals around and inside the left nodules were extremely abundant

**Fig. 2:**
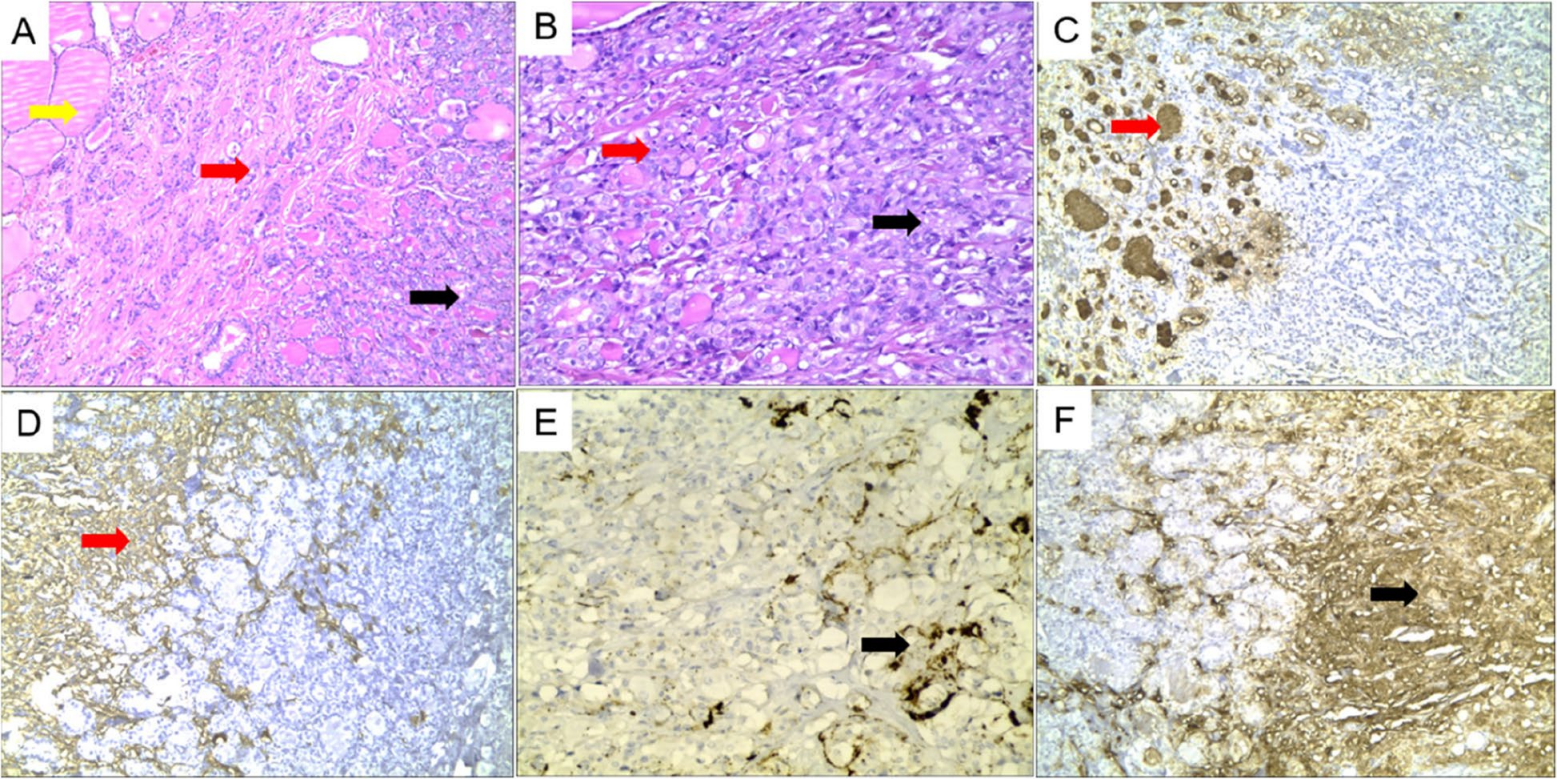
Pathological findings of thyroid collision carcinoma. **A** HE staining showed mixed presence of normal thyroid tissue (yellow arrow), papillary carcinoma (red arrow) and medullary carcinoma (black arrow) at low power, and the two cancers showed crossover (40 ×). **B** Papillary thyroid cancer cells were arranged in follicular shape, with increased nucleus-to-plasma ratio, ground hyaline nucleus, and nuclear furrow (red arrow). Medullary thyroid cancer cells were distributed in lamellar nests, and amyloid stroma (black arrow, 200 ×). **C-F** Immunohistochemical staining showed that papillary thyroid carcinoma was positive for TG (**C**) and CK19 (**D**), while medullary carcinoma was negative. On the contrary, CT (**E**) and CEA (**F**) of medullary carcinoma was positive, while papillary carcinoma was negative. (100 ×)

Patient 1: A 41-year-old woman presented with a thyroid nodule for 3 days. Palpation examination revealed a right thyroid nodule of 14 mm × 9 mm that was hard and ill-defined, without enlarged lymph nodes on either side of the neck. The surgeon suspected thyroid carcinoma. There was no personal history of radiation exposure or family history of thyroid cancer. A change in serum calcitonin was not detected before operating. Color ultrasound revealed a 12 mm × 9 mm hypoechoic nodule with microcalcification in the right thyroid gland, with an ill-defined and uneven internal echo and sandy calcification. No lymph node enlargement was observed on either side of the neck (Fig. 1). The B-scan ultrasound diagnosed thyroid carcinoma with a TIRADS (4a) and the clinical stage was cT1bN0M0. During the operation, the rapid pathological diagnosis showed thyroid carcinoma. The surgeon performed right thyroid lobectomy + left partial thyroidectomy + right neck central lymph node dissection. Postoperative pathological examination showed two histological carcinoma in the nodules of the right lobe. One was a follicular and papillary structure of thyroid with crowded and deformed nuclei, nuclear sulci, nuclear inclusion bodies, and ground glass nuclei. Immunohistochemical examination showed TG ( +), Galectin-3 ( +), CEA (–), and Syn (–). The tumor was diagnosed as papillary thyroid carcinoma (PTC). In the other carcinoma type, round and ovoid cells were segmented into solid patches of nest-like structures by fibrous vascular stroma, with thick chromatin and generally no nucleoli, and amyloid stroma was present. Immunohistochemical results showed TG (–), Galectin-3 (–), CEA ( +), and Syn ( +). The second carcinoma was diagnosed as medullary thyroid carcinoma (MTC; Fig. 2). The two types of carcinomas were close to each other with unclear boundaries. The maximum diameter of the total carcinoma was 1.0 cm and there was lymph node metastasis of the two lymph nodes in the right center neck region. The histological type of metastatic carcinoma in the lymph node was PTC. The pathological stage was pT1bN1M0. After operating, endocrine suppression therapy was applied. The follow-up frequency of the patient was once every 12 months, and there has been no increase in serum calcitonin or CEA and no increase in thyroglobulin to date. Neck ultrasound results showed no abnormalities and there was no recurrence or metastasis at 93 months.

**Fig. 3:**
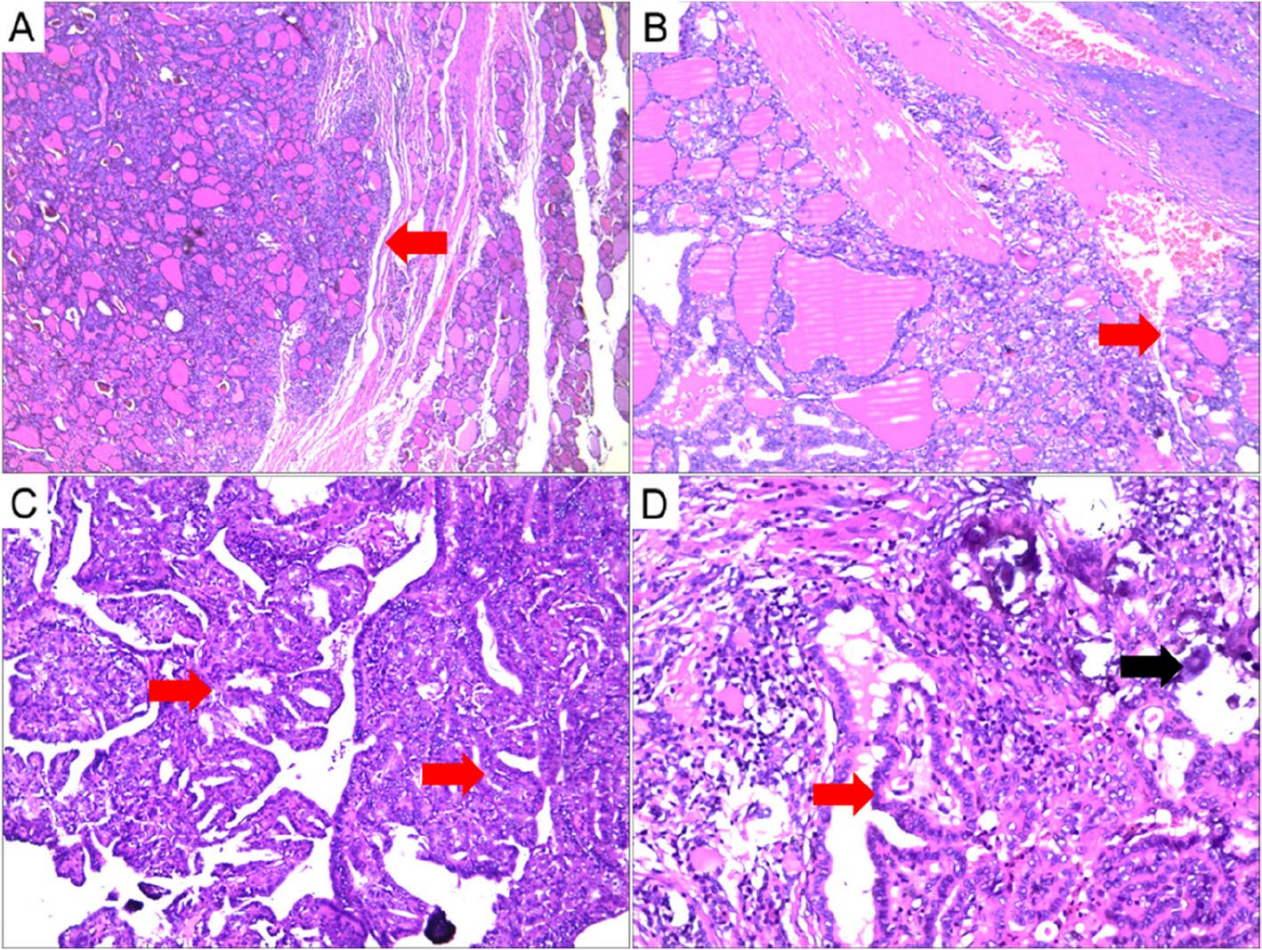
Pathological findings of thyroid composite carcinoma. **A**, **B** Follicular carcinoma of left thyroid showed the cancer cells protruding from the tumor envelope like mushrooms (**A**) with intravascular tumor thrombus (**B**, 40 ×). **C**, **D** Papillary carcinoma of right thyroid presented the cancer cells were arranged in papillary and follicular structures, the cancer cells were tightly arranged and deformed (**C**, 100 ×). The formation of ground-glass nuclei, nuclear furrows, intronuclear inclusions and gritty bodies were observed (**D**, 200 ×)

Patient 2: A 60-year-old woman presented with bilateral thyroid nodules for more than 1 month. Palpation examination revealed a 35 mm nodular lesion in the left and a 17 mm nodular lesion in the right thyroid lobes. The clinical diagnosis was a thyroid tumor. There was no personal history of radiation exposure or family history of thyroid cancer. The serum levels of thyroid hormone and thyroglobulin were normal. Ultrasound showed a solid mass of 35 mm × 29 mm in the left thyroid without calcification and a 26 mm × 18 mm solid mass with calcification in the right thyroid. No lymph node enlargement was observed on either side of the neck (Fig. 1). The B-scan ultrasound diagnosed bilateral benign thyroid nodules with a tumor grade of TIRADS 3. During the operation, a rapid pathological diagnosis was made of follicular carcinoma with a diameter of 3.5 cm in the left thyroid and papillary carcinoma with a diameter of 1.7 cm in the right thyroid. The surgeon performed a total thyroidectomy without neck lymph node dissection. Postoperative histopathology showed that the left thyroid tumor had a follicular structure, the size of the follicles varied, the nuclear-cytoplasmic ratio was slightly increased, the tumor cells were mushroom-shaped with a protruding tumor envelope invading the surrounding normal thyroid gland, and the intravascular tumor thrombus was visible. The tumor was diagnosed as follicular thyroid carcinoma (FTC). In the right thyroid tumor, papillary, acinar-like structures and gritty bodies were found. The nuclear-cytoplasmic ratio was increased, and nuclear sulci, intranuclear inclusion bodies, and ground glass nuclei were visible. The carcinoma in the right thyroid was diagnosed as PTC (Fig. 3). The pathological stage of the left thyroid carcinoma was pT2N0M0 and that of the right was pT1N0M0. Postoperative endocrine suppression therapy was performed without radioiodine therapy. We followed up every 12 months and measured serum HTG and performed a body bone scan, chest CT, and abdominal and thyroid region lymph node color doppler ultrasound. No lymph node enlargement was found in the postoperative follow-up at 60 months and the prognosis was good.

## Discussion

Thyroid cancer is a common malignant tumor and its prevalence is increasing globally [4]. The simultaneous occurrence of two different histological types of carcinoma in the thyroid gland is extremely rare. There are three different terms for this phenomenon, namely, mixed carcinoma, compound carcinoma, and collision carcinoma, and they are often used interchangeably. These terms are used to suggest multiple synchronous tumors in the thyroid gland that present as parafollicular- and follicular-derived tumors. Mixed carcinomas and compound carcinomas occur in one lesion. In a mixed carcinoma, the tumor focus is a mix of two different types of cancer cells, while in compound cancer, the two types are closely adjacent. In contrast, collision carcinomas occur in two lesions with normal tissues between them [3]. Compound carcinomas and collision carcinomas have different cell groups, and their pathological characteristics, biological behavior, and clinical characteristics are also different [3]. In this study, the first case was a composite carcinoma and the second case was a collision carcinoma.

Thyroid composite and collision carcinomas usually present as thyroid nodules and some patients also present with other symptoms, including neck pain and shortness of breath [5]. Thyroid composite carcinomas are usually a single nodule while thyroid collision cancer often involves two or more nodules [2, 5, 6]. Serological examination cannot distinguish between composite and collision carcinomas.

The most common pathological types of collision carcinoma and composite carcinoma are PTC and MTC, followed by PTC combined with other thyroid cancer subtypes, and thyroid metastatic collision carcinoma is rare [7]. In our study, the pathologic types of the composite carcinoma were PTC and MTC, and the pathological types in the collision carcinoma were FTC and PTC. It was reported that 61.7% of PTC have the BRAFV600E mutation, which promotes thyroid cancer migration and invasion [8]. In FTC, 17–57% of tumors have RAS gene family mutations, 98% of familial MTCs have RET gene mutations, and 44% of sporadic MTCs have RET gene mutations [9]. Little is known about the genetic changes in thyroid composite carcinomas and collision carcinomas. Among 183 cases of thyroid cancer with both PTC and MTC, 112 cases underwent gene testing and 24 cases (21%) showed a RET gene mutation, five cases had embryogenic mutations, 19 cases had systemic mutations, and the other 8.33% had the V804M gene mutation [10]. Different mutation profiles of RET and BRAF genes were detected in MTC and PTC mixed-tumor foci in different cases, suggesting that the two types of cancer were driven by different genes, indicating the co-occurrence of the two independent cancers [11]. Germ-line point mutations in exons 13 and 14 have been found in cancer tissues with both PTC and MTC [12]. Currently, there are no genetic tests to distinguish between types of cancer foci and the detection of driver genes could be explored to understand the sources of each type of carcinoma and to facilitate the individualized treatment and follow-up of patients.

Because both composite and collision thyroid cancers have two coexisting cancer entities, the clinical stage, histological types, and gene changes of the two types of carcinomas should be considered when formulating treatment plans. Given the rarity and uniqueness of composite or collisional cancers, individualized treatments are recommended for each case. According to the 2015 guidelines of the American Thyroid Association, the basic treatment of MTC is total thyroidectomy and the scope of lymph node dissection is determined based on clinical stage [13]. Surgical treatment is also preferred for FTC and PTC and the scope of surgical resection also depends on the clinical stage. Due to the rarity of both composite and collision thyroid cancers, the guidelines do not specify their treatment. Our first case was a composite thyroid cancer with MTC and PTC. However, due to the rapid pathological examination during the operation, it was not clear whether the PTC was combined with MTC. Surgical treatment was performed according to the diagnosis of PTC with right thyroid lobectomy, left partial thyroidectomy, and right cervical central lymph node dissection. We suggest that the treatment of composite thyroid cancer should include a comprehensive treatment regimen for both types of cancer and be treated according to the most severe tumor type. The standard treatment for thyroid cancer with coexisting MTC and FTC and suspected neck lymph node metastasis is total thyroidectomy plus central or lateral neck lymph node dissection [14]. The role of postoperative radioiodine and medical thyroid suppression is controversial [15]. For kinase inhibitors, targeted therapy may be an option for some patients. New and highly selective drugs, such as LOXO-292, show potential for the treatment of thyroid cancer with RET gene mutations [16, 17] Our second case was a collision cancer of FTC combined with PTC. Some studies have shown that collision tumors are more aggressive than single tumors [18]. However, in the review of Ryan, collision tumors and single tumors are similarly invasive, and it is recommended to treat patients according to the most aggressive tumor of the two [19]. We suggest that the two entities in the collision thyroid cancer should be treated as two separate synchronous primary tumors. As the standard surgical method is total thyroidectomy without left central neck lymph node dissection, it is difficult to accurately stage lymph nodes and to formulate a suitable treatment. No iodine-131 treatment is required.

Whether composite or collision thyroid carcinomas, postoperative monitoring should consider the characteristics of the two types of tumors present. With two different types of pathogenesis, the likelihood of metastasis recurrence may be greater for composite or collision thyroid carcinomas than the single most invasive carcinoma and the risk of blood and lymphatic metastasis may also be higher. However, some studies indicate that the likelihood of metastasis and the prognosis of collision carcinomas are similar to that of a single carcinoma [20]. Due to the small number of cases, an answer to this question cannot currently be given. Common metastatic sites of MTC and PTC are the lung, bone, and liver. The first case of composite thyroid carcinoma had the dual characteristics of MTC and PTC, and underwent subtotal thyroidectomy, with a high probability of postoperative recurrence and metastasis. Therefore, in such cases, a systemic bone scan, abdominal color doppler ultrasound, chest CT, neck ultrasound, neck CT, PET-CT, and other examinations should be performed to evaluate whether the patient has recurrence or metastasis. Because hypothyroidism may give rise to subfertility or infertility, clinicians should give certain treatment for patients with fertility requirements who undergo thyroidectomy [21]. Combined with thyroid function, thyroxine replacement and endocrine suppressive therapy were evaluated to be satisfactory in both humans and animals. The second case of thyroid collision carcinoma involved FTC and PTC, both of which were differentiated thyroid carcinomas and usually have a relatively good prognosis. However, despite the two primary cancers being well-differentiated, collision carcinoma may indicate aggressive behavior and an increased risk of recurrence, as well a greater risk of blood and lymphatic metastasis, than a single tumor [22]. To date, both cases have a good outcome without recurrence or metastasis.

The two patients were satisfied with the surgery effect. However, they were very worried about the recurrence and metastasis of thyroid carcinoma. They hope that doctors will pay more attention to the thyroid collision and composite carcinoma through this article. The international standardized consensus in early accurate diagnosis, treatment and prognosis of thyroid collision and composite carcinoma will be achieved as soon as possible.

## Conclusion

Thyroid composite carcinomas and collision carcinomas are extremely rare thyroid tumors. The most common combination is FTC and MTC. For composite carcinomas, it is recommended the cancer is treated according to the most aggressive of the two tumors. In contract, collision carcinomas should be treated as two separate synchronous primary tumors. Considering the complexity of the pathogenesis of composite and collision carcinomas, the follow-up after treatment should be extensive.

## Data Availability

Not applicable.

## Abbreviation

FTC: Follicular thyroid carcinoma
MTC: Medullary thyroid carcinoma
PTC: Papillary thyroid carcinoma
